# Molecular evolution of candidate male reproductive genes in the brown algal model Ectocarpus

**DOI:** 10.1186/s12862-015-0577-9

**Published:** 2016-01-05

**Authors:** Agnieszka P. Lipinska, Els J. M. Van Damme, Olivier De Clerck

**Affiliations:** Phycology Research Group and Center for Molecular Phylogenetics and Evolution, Ghent University, Krijgslaan 281, Building S8, 9000 Ghent, Belgium; Department of Molecular Biotechnology, Laboratory of Biochemistry and Glycobiology, Ghent University, Coupure Links 653, 9000 Ghent, Belgium

**Keywords:** Cell-cell recognition, Gamete receptor, *Ectocarpus*, Brown algae, Fertilization

## Abstract

**Background:**

Evolutionary studies of genes that mediate recognition between sperm and egg contribute to our understanding of reproductive isolation and speciation. Surface receptors involved in fertilization are targets of sexual selection, reinforcement, and other evolutionary forces including positive selection. This observation was made across different lineages of the eukaryotic tree from land plants to mammals, and is particularly evident in free-spawning animals. Here we use the brown algal model species *Ectocarpus* (Phaeophyceae) to investigate the evolution of candidate gamete recognition proteins in a distant major phylogenetic group of eukaryotes.

**Results:**

Male gamete specific genes were identified by comparing transcriptome data covering different stages of the *Ectocarpus* life cycle and screened for characteristics expected from gamete recognition receptors. Selected genes were sequenced in a representative number of strains from distant geographical locations and varying stages of reproductive isolation, to search for signatures of adaptive evolution. One of the genes (Esi0130_0068) showed evidence of selective pressure. Interestingly, that gene displayed domain similarities to the receptor for egg jelly (REJ) protein involved in sperm-egg recognition in sea urchins.

**Conclusions:**

We have identified a male gamete specific gene with similarity to known gamete recognition receptors and signatures of adaptation. Altogether, this gene could contribute to gamete interaction during reproduction as well as reproductive isolation in *Ectocarpus* and is therefore a good candidate for further functional evaluation.

**Electronic supplementary material:**

The online version of this article (doi:10.1186/s12862-015-0577-9) contains supplementary material, which is available to authorized users.

## Background

Sexual selection and the evolution of mating specificity are a central focus to evolutionary biology [1]. Recent advancement in molecular tools allowed for identification of specific genes in several organisms controlling particular stages of male–female interactions at fertilization (e.g., [2, 3]) and with the aid of next-generation sequencing methods we are now closer to understand the evolution of mating specificity [4]. In particular, evolutionary studies of genes involved in recognition between male and female gametes (GRPs) provide important insights into the evolution of reproductive isolation and speciation. It has been shown, that fertilization genes generally evolve faster and are targets of sexual selection, reinforcement, and other evolutionary forces (reviewed in [5–8]. Strength of selection on reproductive genes increases with the potential risk of cross-species hybridization or polyspermy within species, and may be correlated with the rate of adaptive evolution. If progressing genetic isolation would be manifested by reduced fitness or sterility of hybrids, selection acting on the genes involved in fertilization may result in the formation of prezygotic reproductive barriers and consequently enhance probabilities of speciation [9–12].

Signatures of rapid evolution of reproductive genes have been detected across different lineages of the eukaryotic tree, including land-plants [13], insects [14], invertebrates [15, 16] and mammals [17]. For example, genes underlaying fertilization or mating traits in *Drosophila* species showed a lack of evolutionary constraints, suggesting that they were most likely shaped during the early stages of speciation by directional sexual selection [18]. However, complicated mating systems as found in copulating animals or animal-pollinated plants involve synergy of many genetic traits, and make it difficult to disentangle the genetic components under sexual selection. In contrast, marine free-spawning species offer a relatively simple model in which male–female interactions are determined by spatial and temporal factors such as the time and place of gamete release; and sexual selection is narrowed down to the gamete interactions [3]. The latter involve specific recognition processes mediated by peptides or glycoproteins expressed on the surface of sperm or egg (reviewed in [19]) which have been shown to be under strong directional selection (reviewed in [3]). Research conducted on sea urchin bindin, an acrosomal protein binding in a species-specific manner to a receptor on the egg plasma membrane prior to the fusion, is exemplar in this respect (e.g., [16, 20, 21]). Similarly, studies of the sperm receptor lysin and its egg ligand VERL in *Haliotis* revealed a strong excess of nonsynonymous to synonymous divergence (*d*_*N*_*/d*_*S*_) between species driven by positive selection and concerted evolution [22, 23], making it one of the fastest evolving metazoan proteins known. In addition, variable selective pressure exerted on the lysin gene in closely related (ω >1) and more diverged species (ω <1) of abalone suggested, that diversifying selection acts on closely related sympatric species, whereas distantly related species are already relieved from it [15, 24]. This could be indicative of reinforcement [25] where sex involved genes would be under selective pressure to establish barriers to reproduction in reunited populations.

Contrary to the rich literature on GRPs in marine invertebrates, studies identifying sperm-egg recognition proteins and their putative adaptive evolution are relatively scarce in other branches of the eukaryotic tree. Here, we focus on the evolution of genes hypothesized to be involved in sexual reproduction and gamete recognition in the brown algal model *Ectocarpus sp.* (Phaeophyceae, Stramenopiles) [26, 27]. Brown algae present an opportunity to investigate gamete receptors in a lineage, that has been evolving independently from land plants and animals for over a billion years [28, 29]. Similarly to sea urchin and abalone, brown algae represent free spawning species with gamete interaction limited to pheromone signalling and surface recognition. Although proteins involved in fertilization in brown seaweeds have not been described so far, a number of proteins involved in sperm-egg binding were isolated and partially characterized in *Fucus* [30–34]*,* but the underlying genes were never identified. In addition to the studies focusing on *Fucus*, a Sexually Induced Gene 1 (Sig1) in a diatom *Thalassiosira* spp. was shown to have high divergence, both within and between species [35]. Transcription of the gene is upregulated during mating [36], however its exact function is not known. Nevertheless, its extreme divergence indicates a possible function as a barrier to hybridization between geographically distant strains [37, 38]. Homologs of the family of Sig genes have also been found in other Stramenopiles, including *Ectocarpus* [26, 39].

*Ectocarpus* is a cosmopolitan genus composed of three recognized species: *E. siliculosus*, *E. fasciculatus* and *E. crouaniorum*. Nonetheless, cross-fertilization experiments imply that more species exist in reality [40, 41]. Previous experiments suggested that gamete recognition is mediated by N-acetylglucosamine (GlcNAc) residues exposed on the plasma membrane of the female gametes and a lectin-like receptor at the tip of male anterior flagella [42, 43]. However, detailed structural information on the proteins involved and gene sequences for the corresponding receptors are lacking.

**Fig. 1:**
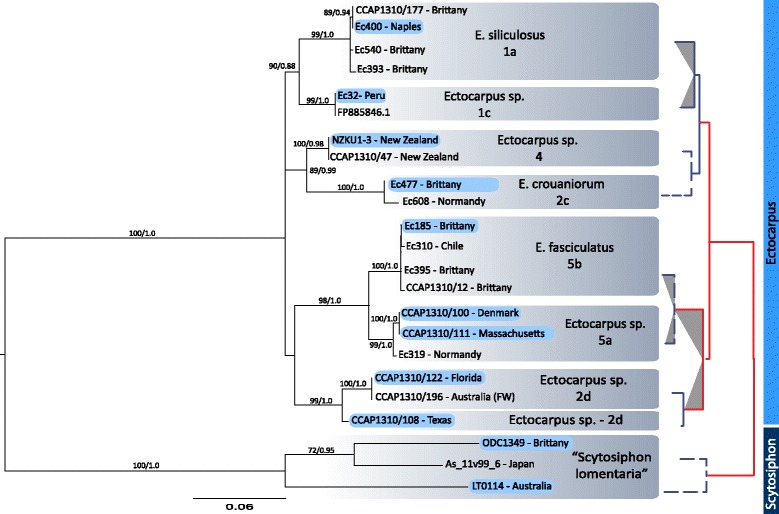
Species tree and species sexual compatibility. Maximum likelihood tree based on the *cox*3 gene from nine *Ectocarpus* strains and two *Scytosiphon lomentaria* specimens. Strains used in this study are marked with blue bars. ML bootstrap (left) and Bayesian posterior probability (right) values are shown. Red lines define species with prezygotic barriers to fertilization; dotted lines describe species with uncertain gamete compatibility; grey shading indicates pairwise *d*_*N*_ */d*_*S*_ > 1 (PAML) (see Additional file 4)

Here, we used large scale genomic data covering different life stages of *Ectocarpus* [44, 45] to identify genes with expression limited to the male gametic stage, expecting surface receptors mediating fertilization to be expressed uniquely in gametes. These genes (Additional file 1) provided a subset to test for divergence and positive selection at the amino acid level using *Ectocarpus* species of known sexual compatibility (Tables 1 and 2, Fig. 1). We found divergence-based evidence of selective pressure acting on at least one of the investigated genes. Interestingly, that gene displayed domain similarities to the receptor for egg jelly (REJ) protein involved in sperm-egg recognition in sea urchins [46].

**Table 1:** Strains used in the study

Lineage	Strain code or species	Short name	Strain no.		Origin
			Ec^a^	CCAP^b^	
4	Ec NZKU 1–3 m	Nze	-	CCAP 1310/56	Kaikoura, New Zealand
5a	Ec Cph 40–11 m	Cph	-	CCAP 1310/100	Copenhagen, Denmark
2d	Ec Tam 2b m	Tam	-	CCAP 1310/122	Tampa, Florida
5a	Ec Pen 2a m	Pen	-	CCAP 1310/111	Penikese Island, Massachusetts
2d	Ec PAr 18a m	Par	-	CCAP 1310/108	Port Aransas, Texas
5b	E. fasciculatus	Fas	Ec185	-	Perharidy, France
1a	E. siliculosus	Nap	Ec400	CCAP 1310/329	Naples, Italy
2c	E. crouaniorum	Cro	Ec477	-	Perharidy, France
1c	Ec 32 (genome strain)	Per	Ec32	CCAP1310/4	San Juan de Marcona, Peru
-	Scytosiphon “lomentaria”	-	ODC1349^c^		Brittany, France
-	Scytosiphon “lomentaria”	-	LT0114^c^		Australia

^a^Macroalgal Culture Collection in Roscoff, France; ^b^Culture Collection of Algae and Protozoa, Oban, Scotland; ^c^deposited at Ghent University, Belgium

**Table 2:** *Ectocarpus* cross-strain fertility

		Male								
		Nap 1a	Per 1c	Cro 2c	Tam 2d	PAr 2d	NZ 4	Cph 5a	Pen 5a	Fas 5b
Female	Nap, 1a	F	post	post	z	z	post	z	pre	post
	Per, 1c	post	F							
	Cro, 2c	post		F	z	z			pre	post
	Tam, 2d	z		z	F	z			pre^a^	
	PAr, 2d	pre		z	z	F			pre^a^	
	NZ, 4	post					F			
	Cph, 5a	z								
	Pen, 5a	pre		pre	pre^a^	pre^a^			F	
	Fas, 5b	pre		pre						F

Data on cross-fertility summarized from [40, 41, 85–87]. Clade numbers corresponding to the phylogenetic position in are given with the strain names. F – full interfertility, z – zygote formation, no data on growth, pre – prezygotic barriers, no cell fusion, post – hybrids with reduced growth or non-functional reproductive structures. ^a^No data on the actual Penikese strain (Pen) are available; fertilization data were inferred from Woods Hole, Massachusetts strain of similar restriction in the mating pattern with other strains, but completely interfertile with the Penikese strain

## Methods

### Selection of genes of interest

Transcriptome data covering male and female gametes [45] as well as gametophyte and sporophyte life stages [44] were compared to obtain a list of male gamete specific genes. An expression filter of RPKM > 1 was applied to remove background noise. Selected genes were further screened for the presence of transmembrane domains using TMHMM [47, 48] and signal peptides targeting the outer membrane using HECTAR [49].

### DNA extraction

DNA was extracted from 20–50 mg of culture material (*Ectocarpus*) or silica dried specimens (*Scytosiphon*). Samples were ground in liquid nitrogen and extracted using CTAB buffer (100 mM Tris–HCl, pH 8.0, 1.4 M NaCl, 20 mM EDTA, 1 % w/v CTAB, 1 % w/v PVP, 0,2 mg/ml proteinase K), followed by two extractions with chloroform:isoamylalcohol (24:1). DNA was precipitated with 80 % isopropanol and cleaned with Qiagen MagAttract Suspension G according to the manufacturer’s instructions.

### Sequence data collection

To perform pairwise divergence analysis, we used genomic data available for the genome strain *Ectocarpus* sp. linage 1c [26, 50] and *Ectocarpus siliculosus* lineage 1a (S. Coelho, unpublished). The two lineages are considered separate ‘species’ with post-zygotic barriers to reproduction (A. Peters, personal communication). Additionally, cultures of nine *Ectocarpus* strains representing clades 1a, 1c, 2c, 2d, 4, 5a and 5b as described by Stache Crain et al. [41] were retrieved from the Culture Collection of Algae and Protozoa (Oban, Scotland) or macroalgal culture collection (Roscoff, France) for DNA extraction. *Scytosiphon* “*lomentaria*” (specimens ODC1349 and LT0114 deposited at Ghent University, Belgium) was used as an outgroup (Fig. 1, Table 1). Primers were designed based on the *Ectocarpus* genome sequence [26, 50] using Primer3 [51, 52] (Additional file 2). Gene fragments were amplified from genomic DNA using a touch-down PCR procedure with initial denaturation for 3 min at 95 °C followed by 10 cycles of denaturation at 95 °C for 30s, 30s annealing at 65 °C decreasing 1 °C per cycle and elongation at 72 °C for 1 min and then 25 cycles of denaturation at 95 °C for 30s, 30s annealing at 55 °C and elongation at 72 °C for 1 min, with a final elongation step of 10 min at 72 °C. Amplicons were sequenced using Sanger sequencing. DNA chromatograms were edited and checked using BioNumerics (Applied Maths). Sequences can be retrieved under accession numbers LN901218 - LN901253. We refer to the Additional file 3 for full details.

### Phylogenetic analyses

Sequences of the mitochondrial cytochrome oxidase subunit 3 (*cox*3) were generated for phylogenetic analysis. Obtained sequences were compared with *Ectocarpus* sequences in GenBank using blastn [53]. Since Bayesian inference and maximum likelihood methods may produce conflicting results, gene sequences were analyzed using both methodologies. Maximum likelihood analyses were carried out with RAxML version 7.7.1 [54] on the RAxML blackbox server (http://phylobench.vital-it.ch/raxml-bb/) using the CAT model [55]. Searches were started from 200 distinct randomized maximum parsimony starting trees and branch support was assessed with the classic bootstrapping algorithm (1000 replicates). Bayesian phylogenetic inference was carried out with MrBayes version 3.2 [56]. Two independent runs, each consisting of four incrementally heated chains, were run for 3 million generations using default priors and other settings. Trees were sampled every thousand generations. Convergence of likelihood and parameter values were assessed with Tracer version 1.5 [57] and a suitable burn-in value was chosen (burnin = 500). Bayesian posterior probabilities for clades were computed from the post burn-in sample of trees and indicated on the Maximum Likelihood (ML) tree (Fig. 1).

### Divergence and positive selection analyses

To estimate the rates of evolution of putative male reproductive genes (receptors), we performed a pairwise *d*_*N*_*/d*_*S*_ analysis using coding sequences of *Ectocarpus* sp. genome strain lineage 1c and *Ectocarpus siliculosus* lineage 1a [41]. Orthologous sequences were aligned using ClustalW implemented in MEGA6 [58, 59] and manually curated. A pairwise *d*_*N*_*/d*_*S*_ (ω) analysis was performed using Phylogenetic Analysis by Maximum Likelihood (PAML, CODEML, F3x4 model, runmode = −2) [60].

Positive selection was estimated by the maximum likelihood method available in CODEML, PAML 4 using the F3X4 model of codon frequencies and paired nested site models of sequence evolution (M0, M3; M1a, M2a; M7, M8) [24, 61, 62]. Nested models were compared using the likelihood ratio test (LRT) with either 2 (M0, M3) or 4 degrees of freedom (M1a,M2a; M7, M8). Empirical Bayes methods allowed for identification of positively selected sites a posteriori [60, 62]. Codon-based nucleotide alignments were used in conjunction with the species tree (see above). Individual exon sequences representing the same gene for a given strain were concatenated using FaBox [63].

Since signatures of positive selection might be overlooked due to its transient or episodic nature, we performed, in parallel, an analysis of occasional selection at individual sites using MEME (Mixed Effects Model of Evolution), implemented in HyPhy [64] available at Datamonkey.org server [65, 66]. The F81 codon substitution model was used in HyPhy [67].

### Protein functional characteristics prediction

Protein structure prediction was done using Phyre2 (Protein Homology/Analogy Recognition Engine) [68]. Transmembrane helices and their topology were inferred from memsat-svm implemented in Phyre2 or from the TMHMM server v. 2.0 [47, 48]. To determine functional domains we performed a Gene Ontology (GO) and protein domain search (InterPro database) using Blast2GO v. 2.6.6 [69] with an E-value Hit Filter set to 1.0e-6.

## Results

### Phylogeny

Maximum likelihood and Bayesian analyses of the mitochondrial *cox*3 gene dataset yielded essentially the same phylogenetic trees (Fig. 1), which divided *Ectocarpus* into 4 well-supported clades. The relationships among these clades, however, remained unresolved. Each clade is subdivided into subclades, again with near full support. While some of these subclades bear formal taxonomic names (e.g., *E. crouaniorum, E. fasciculatus* and *E. siliculosus*), others are known by informal identifiers only. Major clades are reproductively isolated by prezygotic barriers. For subclades such data are either not available or no prezygotic barriers were identified by means of no-choice experiments, however, post-zygotic barriers may exist (see Table 2).

### Screening for candidate reproductive genes

Comparative analysis of transcriptomic data of male and female gametes [45], male and female immature gametophytes and sporophyte of *Ectocarpus* [44] identified 109 male gamete specific genes. These were investigated further for the presence of signal peptides, transmembrane helixes and functional domains potentially involved in cell-cell recognition. Fertilization experiments in *Ectocarpus* have shown the presence of lectin-like receptors in male gametes localized on the anterior flagellum [42]. Therefore, we restricted the search to male expressed genes coding for extracellular or cell surface proteins with potential receptor activity. With these criteria, twelve out of 109 genes were identified as putative gamete recognition genes and were selected for evolutionary analyses together with twelve house-keeping genes as controls (Additional file 4). In addition, we included three genes belonging to the Sig family (Sexually induced genes), previously described as potential recognition genes in diatoms [35]. The *Ectocarpus* genome codes for three Sig-like proteins, two of which showed no expression in gametes (Esi0033_0091 and Esi0116_0079) and one homolog of Sig1 (Esi0101_0018) which was found abundant in both male and female gametes. It has to be noted, that during the initial phase of gamete release both male and female gametes are flagellated in *Ectocarpus*. Therefore, the presence of Sig1 in both sexes may indicate a structural role rather than the involvement in gamete recognition as hypothesized by Honda et al. [39] and Blackman et al. [70].

### Evolution of putative male receptors

To test for evolutionary divergence of candidate male receptor genes, we calculated levels of nonsynonymous (*d*_*N*_) and synonymous (*d*_*S*_) substitution using pairwise comparisons between *Ectocarpus* sp. (lineage 1c) and its sister species *Ectocarpus siliculosus* (lineage 1a). Overall putative receptor genes showed significantly faster evolutionary rates (*d*_*N*_*/d*_*S*_) compared to housekeeping genes (*U* test, *p* = 0.006), with Sig genes showing the highest sequence conservation among putative receptors. Six out of twelve selected male specific genes showed *d*_*N*_*/d*_*S*_ ratio >0.5, which could imply adaptive evolution (Table 3, *d*_*N*_*/d*_*S*_ >0.5 marked in bold). It has been shown that the gene length may bias the estimations of *d*_*N*_*/d*_*S*_ particularly for short genes with low divergence which are then preferably found to be under positive selection [71]. However, higher *d*_*N*_*/d*_*S*_ in this study were associated with GOIs, which were on average substantially longer than housekeeping genes (720 bp vs 358 bp, respectively). We therefore believe that the gene length does not have a major influence on the evolutionary rates of the genes analyzed here.

**Table 3:** Parameter estimates under pairwise sequence analysis in CODEML, PAML 4 using *Ectocarpus siliculosus* lineage 1a vs *Ectocarpus* sp. lineage 1c

Gene	Type^a^	Alignment length (AA)	ω = *d*_*N*_ */d*_*S*_	*d*_*N*_	*d*_*S*_	κ
Esi0018_0186	GOI	513	0.154	0.016	0.107	5.66
Esi0026_0077	GOI	310	0.596	0.034	0.057	2.45
Esi0030_0082	GOI	975	0.435	0.041	0.094	4.33
Esi0033_0091	GOI	184	0.106	0.004	0.040	9.69
Esi0043_0064	GOI	679	0.387	0.029	0.074	2.89
Esi0101_0018	GOI	727	0.025	0.005	0.190	2.82
Esi0116_0079	GOI	375	0.049	0.006	0.123	7.02
Esi0130_0068	GOI	1349	0.529	0.022	0.042	2.09
Esi0132_0081	GOI	1464	0.577	0.048	0.083	2.64
Esi0146_0068	GOI	1537	0.705	0.041	0.058	2.86
Esi0180_0035	GOI	410	0.499	0.026	0.052	2.79
Esi0183_0054	GOI	428	0.338	0.024	0.071	2.76
Esi0186_0062	GOI	687	0.255	0.025	0.098	4.69
Esi0188_0041	GOI	429	0.137	0.011	0.081	2.34
Esi0660_0004	GOI	745	0.533	0.038	0.071	2.05
Esi0008_0135	HKG	533	0.214	0.021	0.098	2.565
Esi0010_0097	HKG	316	0.136	0.0099	0.0733	4.154
Esi0010_0133	HKG	363	0.157	0.0122	0.0777	3.494
Esi0021_0112	HKG	228	0.192	0.0125	0.065	1.986
Esi0044_0085	HKG	319	0.035	0.0037	0.1052	5.886
Esi0069_0059	HKG	609	0.108	0.0106	0.0981	3.173
Esi0116_0065	HKG	92	0	0	0.2612	2.829
Esi0138_0009	HKG	642	0.002	0.0006	0.3315	1.414
Esi0159_0021	HKG	375	0.079	0.0065	0.0814	5.385
Esi0197_0055	HKG	89	0.479	0.0231	0.0483	0.645
Esi0289_0026	HKG	372	0	0	0.0777	2.165
Esi0387_0021	HKG	435	0.016	0.0017	0.1097	1.215

^a^GOI - gene of interest; HKG - housekeeping gene

Esi0130_0068 was particularly interesting due to the presence of a REJ-like domain (IPR002859). REJ-like domains are found in the sperm proteins of sea urchins, where they mediate egg-sperm binding [21, 46]. The remaining genes had either no functional domains (Esi0180_0035), could be involved in substrate transport (Esi0026_0077), lipid metabolism (Esi0132_0081, Esi0146_0068) or nucleic acid hydrolysis (Esi0660_0004) (Additional file 4). We therefore selected 130_0068, sig1 and a couple of house-keeping genes for sequencing in the 9 *Ectocarpus* strains to search for signatures of adaptive evolution using maximum likelihood method implemented in CODEML, PAML 4 [60] and HyPhy [64]. With this approach we obtained evidence that Esi0130_0068 is evolving under positive selection (Tables 4 and 5).

**Table 4:** Positively selected sites identified by the site-prediction methods in PAML 4 and HyPhy (DATAMONKEY)

		Pr > 90 %		*p* value <0,1
Gene	ω = *d*_*N*_ */d*_*S*_^a^	M1a-M2a	M7-M8	MEME
Esi0130_0068	0.7	155	155	224, 230, 303, 626, 819, 820, 823
Esi0101_0018	0.1	none	none	none
Esi0289_0026	0	none	none	none
Esi0069_0059	0	none	none	none
Esi0138_0009	0	none	none	none

^a^Estimate of *d*_*N*_ */d*_*S*_ assuming no rate heterogeneity (model M0, CODEML, PAML4)

**Table 5:** Likelihood ratio statistics (2delta L) for Esi0130_0068

Comparison	2delL	df	Chi-squared 5 %	Chi-squared 1 %
M0 (one ratio) vs. M3 (discrete)	15.229618	4	3753	13,28
M1a (nearly neutral) vs. M2a (positive selection)	6.603632	2	5,99	9,21
M7 (beta) vs. M8 (beta&w)	6.723490	2	5,99	9,21

### Interspecies polymorphism and evidence for positive selection in Esil0130_0068

**Fig. 2:**
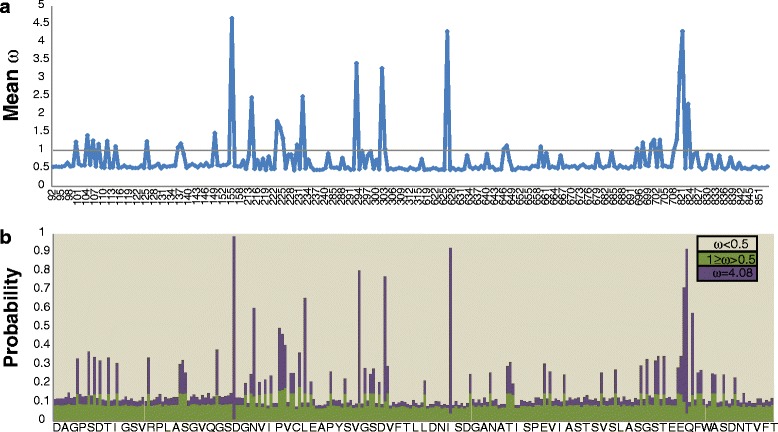
Posterior means of ω and probabilities for site classes in Esi0130_0068. **a** Posterior probabilities for site classes in Esi0130_0068 as calculated by M8 (beta&ω) model in PAML. The ω ratios are 0.00000, 0.00005, 0.00618, 0.12717, 0.64633, 0.96054, 0.99825, 0.99997, 1.00000, 1.00000 and 3.55267. Each of the first 10 categories has a proportion of 0.08717 and the last category has a proportion of 0.12826. Categories are grouped by ω < 0.65, 1 ≥ ω > 0.96, ω = 3.55. **b** Posterior means of ω, calculated as the average over 11 site classes. The amino acid sequence represents the genome strain (Ec32), the site numbers correspond to the position in the coding sequence

**Fig. 3:**
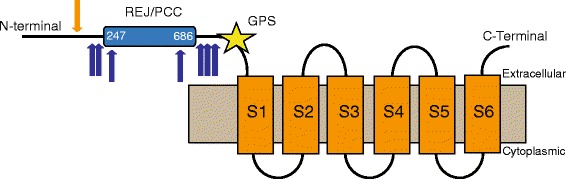
Topology of Esi0130_0068 as predicted by Phyre2 and Blast2GO. The extracellular and cytoplasmic sides of the membrane are labeled and the beginning and end of each transmembrane helix are indicated by the residue index. Positively selected sites identified by PAML 4 are marked with orange arrows, sites identified by HyPhy are marked in blue

The Esi0130_0068 protein consists of 1427 amino acids and presumably contains 7 (TMHMM algorithm) or 6 (memsat-svm algorithm) transmembrane domains (Fig. 2). The N-terminal region, composed of 976 amino acids, is predicted to be located extracellularly. A functional domain scan for this part of the protein identified a GPS domain (*E*-values = 3.78e-05), a REJ domain (*E*-value = 3.98e-30) and a polycystin cation channel (Na^+^, K^+^, Ca^2+^ channel activated by Ca^2+^) within the REJ domain (*E*-value = 3.01e-05). The N-terminal fragment was targeted for resequencing in representative *Ectocarpus* strains. Esi0130_0068 showed statistical evidence for adaptive evolution in the PAML 4 and MEME (HyPhy) analysis. However, the latter analysis presented different sites under selection depending on the model used (Table 4). All models allowing individual sites to evolve under positive selection (M3, M2a, M8) gave a significantly better fit to the Esi0130_0068 data (Table 5) and identified a substantial proportion of sites with *d*_*N*_*/d*_*S*_ >1 (Fig. 2). This result is consistent with an evolutionary history characterized by frequent episodes of positive selection. All three models (M3, M2a, M8) suggest ~7 % of sites under positive selection with ω_2_ = 4.07 (Table 6). The codons inferred to be under positive selection by PAML 4 with posterior probability >90 % lie within the REJ domain in the extracellular region of the protein. Sites identified by HyPhy are adjacent to the N-terminal site of the REJ domain (Fig. 3) and are also indicated by the Empirical Bayes analysis in PAML 4 with lower probability. Noteworthy, sequence analysis of sperm PKDREJ in primates also revealed several positively selected sites in the REJ domain and its flanking extracellular regions [72].

**Table 6:** Parameter estimates and log-likelihood values under models of variable w ratios among sites for Esi0130_0068

Model	*p*	Parameters	*l*	*d*_*N*_ */d*_*S*_	Positively selected sites BEB (Pr > 90 %)
M0: one ratio	1	ω = 0.65860	–1812.54	=ω	None
M1a: nearly neutral	2	p_0_ = 0.45184, ω_0_ = 0.08242, p_1_ = 0.54816, ω_1_ = 1.00000	–1808.23	0.5854	Not allowed
M2a: positive selection	4	p_0_ = 0.93134, ω_0_ = 0.45793, p_1_ = 0.00000, ω_1_ = 1.00000, p_2_ = 0.06866, ω_2_ = 4.06616	–1804.93	0.7057	155
M3: discrete	5	p_0_ = 0.88395, ω_0_ = 0.45793, p_1_ = 0.04738, ω_1_ = 0.45796, p_2_ = 0.06866, ω_2_ = 4.06615	–1804.93	0.7057	155, 626, 821
M7: beta	2	p = 0.02684 q = 0.01590	–1808.29	0.6092	Not allowed
M8: beta& ω	4	p_0_ = 0.93179 p = 83.98499 q = 99.00000, (p_1_ = 0.06821) ω = 4.07807	–1804.93	0.7058	155

## Discussion

Adaptive evolution has commonly been observed in proteins responsible for egg-sperm interactions (for a review see [5]) with a particularly large proportion of positively selected sites found in the sperm-egg binding moieties [6, 73–75]. This indicates that gamete recognition might be subject to selective pressure and sexual selection may operate at the gamete level [5].

Although proteins responsible for egg-sperm interactions in brown algae have not been identified so far, Sig1 was originally hypothesized to play a role in gamete adhesion in the diatom *Thalassiosira* [35, 36]. Sig1 shows high nonsynonymous sequence divergence between closely related species of *Thalassiosira* [35]. Although *Ectocarpus* possesses one homolog of Sig1 with similar length and domain architecture, no evidence was obtained for positive selection in the C-terminal region (amino acids 514–721, exons 5–6). Taken into account that Sig1 is expressed in both types of gametes, these findings support the structural role as mastigoneme proteins rather than their involvement in gamete recognition. It should be noted that the remaining exons (1–4), that might bear the positively evolving sites, could not be amplified by PCR from the genomic DNA and thus are missing in this analysis.

One of the genes expressed specifically in male gametes (Esi0130_0068) revealed significant variation in selective pressure acting on different amino acids. The changes in the evolutionary rates could be a result of weaker selective constrains acting on Esi0130_0068 and/or positive selection. The former could be caused by gene expression bias, if deleterious alleles are less effectively removed when expressed only by one sex in the population [76]. Therefore, gene expression bias could result in relaxation of purifying selection on protein-coding genes. This phenomenon is common in genes with sex-biased expression ([77, 78]; reviewed in [79]) and has been observed also in *Ectocarpus* [44]. The relaxed selective constrain may also facilitate specialized adaptation [76, 80]. To test for signatures of adaptive evolution in the genes of interest, we performed a maximum likelihood test using PAML [60] and HyPhy [66]. Both methods found that divergence in Esi0130_0068 sequence is promoted by adaptive evolution. Analysis of *d*_*N*_*/d*_*S*_ ratio among individual sites identified particular amino acids with good statistical support of positive selection (Table 6), indicative of their putative importance for the function of Esi0130_0068.

Pairwise *d*_*N*_*/d*_*S*_ analysis of Esi0130_0068 showed highest values (ω > 1) in comparisons between closely related strains, which concerns clades 5a, 5b and 2d as well as clades 1a and 1c (Additional file 5, Fig. 1). While pre-zygotic barriers to fertilization already exist between clades 5a and 2d, clades 1a and 1b are able to form zygotes which are later arrested during development (Table 2). In this case, excess of nonsynonymous to synonymous substitutions could be a sign of diversifying selection acting to reinforce reproductive barriers between strains 1a and 1c. Similar scenario could account for the high diversity between clades 5a and 5b, however compatibility studies are lacking in this group.

Interestingly, Esi0130_0068 resembles the topology of the egg recognition protein in sea urchin sperm [46]. Although the exact function of the REJ domain containing proteins is unknown in brown algae the unique localization and expression pattern of REJ proteins in sea urchins and humans (hPKDREJ) suggest a central role in fertilization [46, 81]. Sea urchin sperm contains several members of the REJ protein family (named SpREJ1-10), out of which SpREJ1 binds a fucose sulfate polymer of the egg jelly, triggering the acrosome reaction and transforming the sperm into a fusogenic cell [21, 46, 82]. *Ectocarpus* Esi0130_0068 protein does not contain the lectin domain upstream of REJ, which probably interacts with the female gamete surface glycoprotein in sea urchin, however this domain is also not found in the human hPKDREJ. In sea urchins, the carbohydrate recognition domains that are subjected to positive selection [75] in contrast to the sperm PKDREJ in primates were positively selected sites are found within and around the REJ domain [72]. It is unclear at present whether the REJ domain itself can take part in the interaction with the egg glycoprotein coat The REJ domain has the potential role in regulating the ion channel which triggers the acrosome reaction [81, 83]. Therefore, the REJ domain may be important for triggering the species-specific recognition cascade through control of the ion flux. Additionally, almost all of the members of the SpREJ and human PKD families possess a G-protein coupled receptor cleavage site (GPS) upstream of the first transmembrane helix [82]. REJ domains are predicted to affect the cleavage at the GPS site [84], which may be another way of influencing the fertilization process. The presence of a REJ domain in combination with a GPS motif and possible cation channel function make Esi0130_0068 an appealing candidate for *in situ* evaluation.

## Conclusions

This study focused on genes for which there is evidence that expression is limited only to male gametes of *Ectocarpus* and possibly subjected to adaptive evolution. By extrapolation, the observed positive selection may pinpoint the genes that are directly involved in male reproduction, which would be an important step towards understanding the molecular basis of gamete interaction during reproduction in *Ectocarpus*. In particular, one male gamete specific gene (Esi0130_0068) appears to be a good candidate due to signatures of positive selection and its similarity to the sperm-egg recognition protein in sea urchin. However, nucleotide sequences used in this study represent only a partial coding sequence of selected genes in a limited number of strains. Future work would require a larger sample size and complete gene sequences for a better estimation of evolution over time and forces shaping the divergence in the sex-related genes in *Ectocarpus*.

## Additional files

Additional file 1:
***Ectocarpus*** **male gamete specific genes.** (XLS 25 kb) Additional file 2:
**Primers used for polymerase chain reaction.** (XLS 8 kb) Additional file 3:
**Sequence accession numbers and details.** (XLS 9 kb) Additional file 4:
**Characteristics of genes of interest used for the pairwise** ***d***_***N***_ ***/d***_***S***_ **analysis.** (XLS 10 kb) Additional file 5:
**Pairwise** ***d***_***N***_ ***/d***_***S***_ **matrix for Esi0130_0068 generated with PAML (runmode = −2).** (XLS 8 kb)

## Abbreviations

GRP: gamete recognition protein
bp: base pair
PCR: polymerase chain reaction
GO: gene ontology
REJ: receptor for egg jelly
GOI: gene of interest
HKG: housekeeping gene
ML: Maximum Likelihood

## References

[1] Bernasconi G, Ashman T-L, Birkhead TR, Bishop JDD, Grossniklaus U, Kubli E (2004). Evolutionary Ecology of the Prezygotic Stage. Science.

[2] Kosman ET, Levitan DR (2014). Sperm competition and the evolution of gametic compatibility in externally fertilizing taxa. Mol Hum Reprod.

[3] Vacquier VD, Swanson WJ (2011). Selection in the Rapid Evolution of Gamete Recognition Proteins in Marine Invertebrates. Cold Spring Harb Perspect Biol.

[4] Hart MW (2012). Next-generation studies of mating system evolution. Evolution.

[5] Clark NL, Aagaard JE, Swanson WJ (2006). Evolution of reproductive proteins from animals and plants. Reproduction.

[6] Clark NL, Gasper J, Sekino M, Springer SA, Aquadro CF, Swanson WJ (2009). Coevolution of Interacting Fertilization Proteins. Plos Genetics.

[7] Swanson WJ, Vacquier VD (2002). Reproductive protein evolution. Annu Rev Ecol Syst.

[8] Turner LM, Hoekstra HE (2008). Causes and consequences of the evolution of reproductive proteins. Int J Dev Biol.

[9] Ferris PJ, Pavlovic C, Fabry S, Goodenough UW (1997). Rapid evolution of sex-related genes in Chlamydomonas. Proc Natl Acad Sci U S A.

[10] Hart MW, Sunday JM, Popovic I, Learning KJ, Konrad CM (2014). Incipient Speciation of Sea Star Populations by Adaptive Gamete Recognition Coevolution. Evolution.

[11] Hellberg ME, Vacquier VD (1999). Rapid evolution of fertilization selectivity and lysin cDNA sequences in teguline gastropods. Mol Biol Evol.

[12] Palumbi SR (2008). Speciation and the evolution of gamete recognition genes: pattern and process. Heredity.

[13] Wang X, Hughes AL, Tsukamoto T, Ando T, Kao T-H (2001). Evidence That Intragenic Recombination Contributes to Allelic Diversity of the S-RNase Gene at the Self-Incompatibility (S) Locus in Petunia inflata. Plant Physiol.

[14] Civetta A, Singh RS (1995). High divergence of reproductive tract proteins and their association with postzygotic reproductive isolation in Drosophila melanogaster and Drosophila virilis group species. J Mol Evol.

[15] Lee YH, Ota T, Vacquier VD (1995). Positive selection is a general phenomenon in the evolution of abalone sperm lysin. Mol Biol Evol.

[16] Metz EC, Palumbi SR (1996). Positive selection and sequence rearrangements generate extensive polymorphism in the gamete recognition protein bindin. Mol Biol Evol.

[17] Clark NL, Swanson WJ (2005). Pervasive Adaptive Evolution in Primate Seminal Proteins. PLoS Genet.

[18] Civetta A, Singh RS (1998). Sex-related genes, directional sexual selection, and speciation. Mol Biol Evol.

[19] Hirohashi N, Kamei N, Kubo H, Sawada H, Matsumoto M, Hoshi M (2008). Egg and sperm recognition systems during fertilization. Develop Growth Differ.

[20] Lessios HA, Lockhart S, Collin R, Sotil G, Sanchez-Jerez P, Zigler KS (2012). Phylogeography and bindin evolution in Arbacia, a sea urchin genus with an unusual distribution. Mol Ecol.

[21] Vacquier VD, Moy GW (1997). The Fucose Sulfate Polymer of Egg Jelly Binds to Sperm REJ and Is the Inducer of the Sea Urchin Sperm Acrosome Reaction. Dev Biol.

[22] Galindo BE, Vacquier VD, Swanson WJ (2003). Positive selection in the egg receptor for abalone sperm lysin. Proc Natl Acad Sci U S A.

[23] Swanson WJ, Vacquier VD (1998). Concerted evolution in an egg receptor for a rapidly evolving abalone sperm protein. Science.

[24] Yang Z (2000). Maximum likelihood estimation on large phylogenies and analysis of adaptive evolution in human influenza virus A. J Mol Evol.

[25] Dobzhansky T (1951). Genetics and the Origin of Species.

[26] Cock JM, Sterck L, Rouzé P, Scornet D, Allen AE, Amoutzias G (2010). The Ectocarpus genome and the independent evolution of multicellularity in brown algae. Nature.

[27] Coelho SM, Scornet D, Rousvoal S, Peters NT, Dartevelle L, Peters AF (2012). Ectocarpus: A Model Organism for the Brown Algae. Cold Spring Harb Protoc.

[28] Cock JM, Coelho SM, Brownlee C, Taylor AR (2010). The Ectocarpus genome sequence: insights into brown algal biology and the evolutionary diversity of the eukaryotes. New Phytol.

[29] Parfrey LW, Lahr DJG, Knoll AH, Katz LA (2011). Estimating the timing of early eukaryotic diversification with multigene molecular clocks. PNAS.

[30] Bolwell GP, Callow JA, Callow ME, Evans LV (1979). Fertilization in brown algae. II. Evidence for lectin-sensitive complementary receptors involved in gamete recognition in Fucus serratus. J Cell Sci.

[31] Bolwell GP, Callow JA, Evans LV (1980). Fertilization in brown algae. III. Preliminary characterization of putative gamete receptors from eggs and sperm of Fucus serratus. J Cell Sci.

[32] Callow JA, Stafford CJ, Green JR: Gamete recognition and fertilisation in the fucoid algae. In *Perspectives in Plant Cell Recognition*. Cambridge University Press; 1992. [*Society for Experimental Biology Seminar Series*].

[33] Wright PJ, Green JR, Callow JA (1995). The Fucus (phaeophyceae) Sperm Receptor for Eggs. I. Development and Characteristics of a Binding Assay1. J Phycol.

[34] Wright PJ, Callow JA, Green JR (1995). The Fucus (phaeophyceae) Sperm Receptor for Eggs. Ii. Isolation of a Binding Protein Which Partially Activates Eggs1. J Phycol.

[35] Armbrust EV, Galindo HM (2001). Rapid evolution of a sexual reproduction gene in centric diatoms of the genus Thalassiosira. Appl Environ Microbiol.

[36] Armbrust EV (1999). Identification of a new gene family expressed during the onset of sexual reproduction in the centric diatom Thalassiosira weissflogii. Appl Environ Microbiol.

[37] Sorhannus U (2003). The effect of positive selection on a Sexual Reproduction Gene in Thalassiosira weissflogii (Bacillariophyta): results obtained from maximum-likelihood and parsimony-based methods. Mol Biol Evol.

[38] Sorhannus U, Kosakovsky Pond S (2006). Evidence for positive selection on a Sexual Reproduction Gene in the diatom genus Thalassiosira (Bacillariophyta). J Mol Evol.

[39] Honda D, Shono T, Kimura K, Fujita S, Iseki M, Makino Y (2007). Homologs of the Sexually Induced Gene 1 (sig1) product constitute the Stramenopile mastigonemes. Protist.

[40] Peters AF, van Wijk SJ, Cho GY, Scornet D, Hanyuda T, Kawai H (2010). Reinstatement of Ectocarpus crouaniorum Thuret in Le Jolis as a third common species of Ectocarpus (Ectocarpales, Phaeophyceae) in Western Europe, and its phenology at Roscoff, Brittany. Phycol Res.

[41] Stache Crain B, Muller DG, Goff LJ (1997). Molecular systematics of Ectocarpus and Kuckuckia (Ectocarpales, Phaeophyceae) inferred from phylogenetic analysis of nuclear- and plastid-encoded DNA sequences. J Phycol.

[42] Schmid CE (1993). Cell-cell-recognition during fertilization in Ectocarpus siliculosus (Phaeophyceae). Hydrobiologia.

[43] Schmid CE, Schroer N, Muller DG (1994). Female gamete membrane glycoproteins potentially involved in gamete recognition in Ectocarpus siliculosus. Plant Sci.

[44] Lipinska A, Cormier A, Luthringer R, Peters A, Corre E, Gachon C (2015). Sexual dimorphism and the evolution of sex-biased gene expression in the brown alga Ectocarpus. Mol Biol Evol.

[45] Lipinska AP, D’hondt S, Damme EJV, Clerck OD (2013). Uncovering the genetic basis for early isogamete differentiation: a case study of Ectocarpus siliculosus. BMC Genomics.

[46] Moy GW, Mendoza LM, Schulz JR, Swanson WJ, Glabe CG, Vacquier VD (1996). The sea urchin sperm receptor for egg jelly is a modular protein with extensive homology to the human polycystic kidney disease protein, PKD1. J Cell Biol.

[47] Krogh A, Larsson B, von Heijne G, Sonnhammer ELL (2001). Predicting transmembrane protein topology with a hidden Markov model: Application to complete genomes. J Mol Biol.

[48] Sonnhammer EL, von Heijne G, Krogh A (1998). A hidden Markov model for predicting transmembrane helices in protein sequences. Proc Int Conf Intell Syst Mol Biol.

[49] Gschloessl B, Guermeur Y, Cock JM (2008). HECTAR: A method to predict subcellular targeting in heterokonts. BMC Bioinformatics.

[50] Sterck L, Billiau K, Abeel T, Rouzé P, Van de Peer Y (2012). ORCAE: online resource for community annotation of eukaryotes. Nat Meth.

[51] Koressaar T, Remm M. Enhancements and modifications of primer design program Primer3. Bioinformatics. 2007;23:1289–91. 10.1093/bioinformatics/btm091 17379693

[52] Untergasser A, Cutcutache I, Koressaar T, Ye J, Faircloth BC, Remm M (2012). Primer3--new capabilities and interfaces. Nucleic Acids Res.

[53] Altschul SF, Madden TL, Schäffer AA, Zhang J, Zhang Z, Miller W (1997). Gapped BLAST and PSI-BLAST: a new generation of protein database search programs. Nucleic Acids Res.

[54] Stamatakis A, Hoover P, Rougemont J (2008). A rapid bootstrap algorithm for the RAxML Web servers. Syst Biol.

[55] Stamatakis A: Phylogenetic models of rate heterogeneity: a high performance computing perspective. In *Parallel and Distributed Processing Symposium, 2006. IPDPS 2006. 20th International*; 2006:8 pp.–.

[56] Ronquist F, Teslenko M, van der Mark P, Ayres DL, Darling A, Höhna S (2012). MrBayes 3.2: efficient Bayesian phylogenetic inference and model choice across a large model space. Syst Biol.

[57] Rambaut A, Drummond A: *Tracer v1.5, Available from* http://beast.bio.ed.ac.uk/Tracer. 2009.

[58] Larkin MA, Blackshields G, Brown NP, Chenna R, McGettigan PA, McWilliam H (2007). Clustal W and Clustal X version 2.0.. Bioinformatics.

[59] Tamura K, Stecher G, Peterson D, Filipski A, Kumar S (2013). MEGA6: Molecular Evolutionary Genetics Analysis version 6.0. Mol Biol Evol.

[60] Yang Z (2007). PAML 4: Phylogenetic Analysis by Maximum Likelihood. Mol Biol Evol.

[61] Yang Z (1998). Likelihood ratio tests for detecting positive selection and application to primate lysozyme evolution. Mol Biol Evol.

[62] Yang Z, Nielsen R, Goldman N, Pedersen A-MK (2000). Codon-Substitution Models for Heterogeneous Selection Pressure at Amino Acid Sites. Genetics.

[63] Villesen P (2007). FaBox: an online toolbox for fasta sequences. Mol Ecol Notes.

[64] Murrell B, Wertheim JO, Moola S, Weighill T, Scheffler K, Kosakovsky Pond SL (2012). Detecting Individual Sites Subject to Episodic Diversifying Selection. PLoS Genet.

[65] Delport W, Poon AFY, Frost SDW, Kosakovsky Pond SL (2010). Datamonkey 2010: a suite of phylogenetic analysis tools for evolutionary biology. Bioinformatics.

[66] Pond SLK, Frost SDW, Muse SV (2005). HyPhy: hypothesis testing using phylogenies. Bioinformatics.

[67] Felsenstein J (1981). Evolutionary trees from DNA sequences: A maximum likelihood approach. J Mol Evol.

[68] Kelley LA, Sternberg MJE (2009). Protein structure prediction on the Web: a case study using the Phyre server. Nat Protoc.

[69] Conesa A, Gotz S, Garcia-Gomez JM, Terol J, Talon M, Robles M (2005). Blast2GO: a universal tool for annotation, visualization and analysis in functional genomics research. Bioinformatics.

[70] Blackman LM, Arikawa M, Yamada S, Suzaki T, Hardham AR (2011). Identification of a mastigoneme protein from Phytophthora nicotianae. Protist.

[71] Mugal CF, Wolf JBW, Kaj I (2014). Why Time Matters: Codon Evolution and the Temporal Dynamics of dN/dS. Mol Biol Evol.

[72] Hamm D, Mautz BS, Wolfner MF, Aquadro CF, Swanson WJ (2007). Evidence of amino acid diversity-enhancing selection within humans and among primates at the candidate sperm-receptor gene PKDREJ. Am J Hum Genet.

[73] Nydam ML, Harrison RG: Reproductive protein evolution in two cryptic species of marine chordate. BMC Evolutionary Biology. 2011;11:18. 10.1186/1471-2148-11-18 PMC3036616 21247489

[74] Swanson WJ, Yang Z, Wolfner MF, Aquadro CF (2001). Positive Darwinian selection drives the evolution of several female reproductive proteins in mammals. PNAS.

[75] Mah SA, Swanson WJ, Vacquier VD (2005). Positive selection in the carbohydrate recognition domains of sea urchin sperm receptor for egg jelly (suREJ) proteins. Mol Biol Evol.

[76] Mank JE, Ellegren H (2009). Are sex-biased genes more dispensable?. Biol Lett.

[77] Mank JE, Hultin-Rosenberg L, Axelsson E, Ellegren H (2007). Rapid Evolution of Female-Biased, but Not Male-Biased, Genes Expressed in the Avian Brain. Mol Biol Evol.

[78] Gossmann TI, Schmid MW, Grossniklaus U, Schmid KJ (2014). Selection-Driven Evolution of Sex-Biased Genes Is Consistent with Sexual Selection in Arabidopsis thaliana. Mol Biol Evol.

[79] Ellegren H, Parsch J (2007). The evolution of sex-biased genes and sex-biased gene expression. Nat Rev Genet.

[80] Chapman T (2006). Evolutionary Conflicts of Interest between Males and Females. Curr Biol.

[81] Hughes J (1999). Identification of a human homologue of the sea urchin receptor for egg jelly: a polycystic kidney disease-like protein. Hum Mol Genet.

[82] Gunaratne HJ, Moy GW, Kinukawa M, Miyata S, Mah SA, Vacquier VD (2007). The 10 sea urchin receptor for egg jelly proteins (SpREJ) are members of the polycystic kidney disease-1 (PKD1) family. BMC Genomics.

[83] Trimmer JS, Schackmann RW, Vacquier VD (1986). Monoclonal antibodies increase intracellular Ca2+ in sea urchin spermatozoa. PNAS.

[84] Qian F, Boletta A, Bhunia AK, Xu H, Liu L, Ahrabi AK (2002). Cleavage of polycystin-1 requires the receptor for egg jelly domain and is disrupted by human autosomal-dominant polycystic kidney disease 1-associated mutations. PNAS.

[85] Müller DG (1979). Genetic affinity of Ectocarpus siliculosus (Dillw.) Lyngb. from the Mediterranean, North Atlantic and Australia. Phycologia.

[86] Stache B (1990). Sexual compatibility and species concept in Ectocarpus siliculosus (Ectocarpales, Pheophyceae) from Italy, North Carolina, Chile, and New Zealand. Evolutionary biogeography of the marine algae of the North Atlantic.

[87] Muller DG, Eichenberger W (1995). Crossing experiments, lipid composition, and the species concept in Ectocarpus siliculosus and E. fasciculatus (Pheophyceae, Ectocarpales). J Phycol.

